# Analytical validation of the Belay Vantage^™^ assay for evaluation of *MGMT* promoter methylation using enzymatically converted tumorDNA from cerebrospinal fluid

**DOI:** 10.1016/j.cancergen.2025.04.001

**Published:** 2025-04-05

**Authors:** Kala F Schilter, Qian Nie, Jennifer N Adams, Rakshitha Jagadish, Anthony Acevedo, Alexandra Larson, Samantha A Vo, Brett A Domagala, Kyle M Hernandez, Christopher Douville, Yuxuan Wang, Brian Coe, Chetan Bettegowda, Honey V Reddi

**Affiliations:** Belay Diagnostics, Suite 530, 1375W. Fulton St, Chicago, IL 60607, USA

**Keywords:** *MGMT* methylation, Enzymatic conversion, Cerebrospinal fluid, Liquid biopsy, Quantitative PCR

## Abstract

*MGMT* promoter methylation status (hypermethylation) is one of the strongest prognostic and predictive biomarkers in glioblastoma (GBM) and is associated with a more favorable response to alkylating chemotherapies such as Temozolomide (TMZ). Additionally, it is associated with pseudo progression in GBM, a phenomenon in which early radiographic changes after treatment are indicative of possible tumor recurrence though on histological examination it is consistent with treatment effect. Current methods for evaluation of *MGMT* promoter methylation status are limited to tumor tissue, requiring invasive biopsy or surgery, prompting the need for a liquid biopsy-based assay to expand and manage therapeutic interventions. The Belay Vantage^™^ assay evaluates *MGMT* promoter methylation status in cerebrospinal fluid (CSF) of individuals with known or suspected central nervous system tumors using low input DNA. The assay uses quantitative polymerase chain reaction (qPCR) on DNA extracted from CSF after enzymatic conversion and has an analytical sensitivity of 95.5 % and specificity of 100 %.

## Introduction

Current standard of care for diagnosis and detection of CNS tumors includes magnetic resonance imaging (MRI) which can be non-specific [1,2], and CSF Cytology which requires substantial volume (>10 mL) of CSF and has low sensitivity (41 %) due to the absence of genomic interrogation [3]. Glioblastoma (GBM) also referred to as grade IV astrocytoma is the most common and aggressive brain cancer accounting for 47.7 % of all primary brain malignancies with a prognosis of six months or less, if untreated [4]. While there are several therapeutic options available for primary and metastatic disease [5], therapeutic options for GBM are limited, contributing to the aggressive nature and poor prognosis of this cancer. Methylation of the O6-methylguanine-DNA methyl-transferase *(MGMT)* gene promoter has been observed in approximately 50 % of GBM [6] and guidelines recommend that tumor *MGMT* status be performed with high grade gliomas to possibly expand therapeutic options (NCCN Guidelines Version 2.2022 Central Nervous System Cancers) [7].

*MGMT* promoter methylation status (hypermethylation) is one of the strongest prognostic and predictive biomarkers in GBM [8] and is associated with a more favorable response to alkylating chemotherapies such as Temozolomide (TMZ) [9-11]. Epigenetic modification (methylation) of the CpG island at specific sites within the *MGMT* promoter silences the gene and reduces expression, making tumors more sensitive to alkylating agents [12,13]. Additionally, *MGMT* promoter hypermethylation is associated with pseudo progression, a phenomenon in which early radiographic changes mimic tumor recurrence, when in fact the patient is experiencing treatment effect [14] making this an important biomarker for GBM.

Current commercially available tests to detect methylation of *MGMT* are performed in tumor tissue and utilize bisulfite conversion of the DNA followed by either direct Sanger sequencing, bisulfite-cloning sequencing, pyrosequencing, or methylation-specific PCR (MS-PCR). Treatment of DNA with sodium bisulfite converts unmethylated cytosine to uracil, which is subsequently converted to thymine during PCR amplification, while methyl cytosine remains unchanged. Bisulfite conversion, the current gold standard method of DNA conversion to distinguish methylated from unmethylated cytosines causes damage to the DNA, incomplete genome coverage, incomplete conversion, and DNA fragmentation [15,16]. In contrast to bisulfite conversion, enzymatic conversion does not affect the integrity of the DNA, minimizing damage and increasing the ability to process converted DNA downstream [17].

Recent studies to evaluate *MGMT* as a predictive biomarker in IDH-wildtype GB in liquid biopsy was demonstrated using small extracellular vesicles isolated from blood in comparison to tumor methylation results [18]. Herein we present the analytical validation of the Belay Vantage^™^ assay for evaluation of *MGMT* promoter methylation status in cerebrospinal fluid (CSF) of individuals with known or suspected central nervous system tumors using quantitative polymerase chain reaction (qPCR) evaluation of tumor DNA (tDNA) extracted from CSF after enzymatic conversion.

## Methodology

### Specimen cohort

A total of 60 specimens including 2 oligodendroglioma cell lines (ATCC, USA), BT-54 (CRL-3416) and BT-88 (CRL-3417) 4 contrived controls; HD752 and HD728 (Horizon) and EpiScope Unmethylated HCT116 and EpiScope Methylated HCT116 (Takara Bio), CSF specimens from presumed normal individuals (*n* = 38), CSF from individuals with no diagnosis of cancer (*n* = 3) and suspicion or known diagnosis of cancer (*n* = 7) and DNA from FFPE tumors of individuals with CNS tumors (*n* = 6) were utilized in the validation study. De-identified CSF samples from individuals presumed not to have cancer and deidentified FFPE DNA with variant information were commercially purchased. CSF specimens with known molecular profiling results, diagnoses, and demographic information (age range, sex assigned at birth, ethnicity) were obtained from Johns Hopkins University (JHU) under an institutional IRB (IRB00420181) in compliance with the principles of the Declaration of Helsinki and the Health Insurance Portability and Accountability Act (HIPAA).

### Vantage^™^ assay

**Fig. 1.**
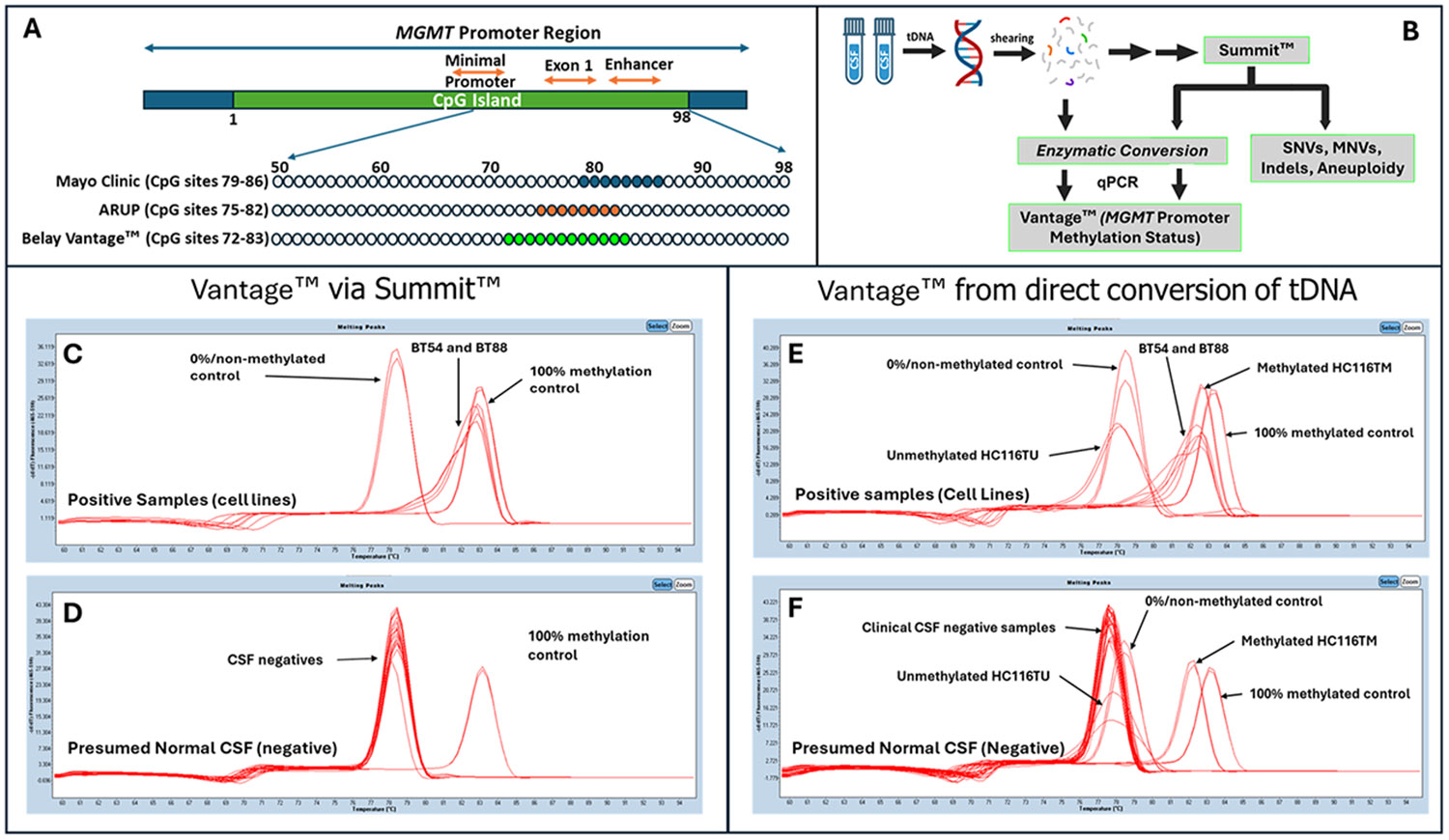
CpG islands evaluated by Vantage^™^ in the *MGMT* promoter region (A). Vantage^™^ workflows directly from tumor derived DNA or via Summit^™^ (B). C-F representative images of the samples aligning with controls in the analysis software; C and E – positive cell lines (BT54 and BT88), D and F – negative CSF samples and contrived controls (HC116TU and HC116TM).

The *MGMT* promoter region includes 98 CpG sites (Fig. 1A). Vantage^™^ evaluates 12 of the 98 CpG sites (#72–83) [19] as shown in Fig. 1A. tDNA extracted from CSF was sheared and either directly subjected to enzymatic conversion (NEBNext Enzymatic Conversion Module, Cat # E7125L) or was converted after library amplification in the Summit^™^ workflow (Fig. 1B). Summit^™^ is a multi-analyte genomic profiling assay [20] that leverages the MethySaferSeqS (MSSS) duplex sequencing previously described [21], to enable robust detection of variants in CSF with low input tumor derived DNA (tDNA) and includes Vantage^™^ as one of its analytes. *MGMT* Promoter Methylation status of the converted tDNA was evaluated via quantitative PCR (qPCR) using the EpiMelt Kit (Cat #: MD-MGMT/EPI-qPCR 200/500) kit (Boca Scientific) followed by high resolution melt analysis. Conversion efficiency was assessed in the unmethylated UMI region (first 14 positions) of MSSS reads by first generating per-cycle base frequencies with DRAGEN (v4.1.23). Efficiency was defined as 1.0 – (C_prop_ / 0.25), where C_prop_ is the proportion of “C”s in the first 14 positions (UMI) and 0.25 is the expected proportion of the C base in this region [21].

Data from qPCR is evaluated using ‘Tm Calling’ algorithm in the LightCycler 480 software (version 1.5.1, Roche LifeScience). Methylated and unmethylated melting temperature peaks are evaluated in direct comparison to the assay controls. The melting peak for the non-methylated control is expected at 78 °C (±2.5 °C) and samples aligning with this control peak are reported as “Negative - No methylation detected”. The melting peak for the methylated control is expected at 83 °C (±2.5 °C). The assay also includes a calibration control (1 % methylated) which is expected at 78 °C and 83 ° C, with a short peak at 83 ° C to evaluate assay sensitivity. Specimens with peaks aligning with the methylated control above the height of the calibration control are interpreted as “Positive - Methylation detected.” Specimens with results between unmethylated and methylated control are interpreted as “Indeterminate – results were equivocal.”

### Analytical validation (AV)

Analytical validation establishes the suitability of a test for its intended purpose by assessing its ability to measure a genetic marker accurately, precisely, and reliably. Demonstration of the accuracy and precision of Vantage^™^ follows ACCE guidelines [22] and includes the determination of limit of Blank (LoB) and limit of input (LoI) for the assay.

## Results

### Limit of blank (LoB) and limit of input (LoI)

To establish the LoB, a total of 10 samples were included, five (5) no template controls (NTCs) and DNA from (5) presumed normal CSF samples with no *MGMT* methylation were processed for Vantage^™^ both directly from sheared DNA and via the Summit workflow. Results demonstrated that no amplification was observed with NTCs and the 5 CSF samples across both workflows aligned with the no methylation controls, demonstrating the absence of noise in the analysis read-out. To determine the lowest input for the Vantage^™^ assay, different concentrations of DNA (5 ng, 10 ng, 15 ng, 20 ng and 40 ng) from four (4) contrived controls were evaluated through both workflows, directly from sheared DNA and via Summit^™^. All samples at different input yielded concordant results demonstrating the lowest input of Vantage^™^ from both workflows to be 5 ng.

### Analytical accuracy of vantage^™^

Accuracy of Vantage^™^ via the Summit^™^ workflow was evaluated using 58 samples with known *MGMT* status. Methylation conversion efficiency was high among samples (95.07 % ± 2.04 %). Both contrived controls and cell lines showed positive methylation as expected (Fig. 1C) and all 38 presumed normal CSF were negative as expected (Fig. 1D). All 6 FFPE samples and nine of 10 cancer CSF specimens demonstrated concordant results (Table 1). One of the CSF samples resulted as negative result for a previously reported positive possibly due to tumor heterogeneity in what metastasized and differences in tests being used for evaluation (technical variation),establishing the sensitivity of Vantage^™^ via the Summit^™^ to be 91 % with a specificity of 100 %.For the direct conversion accuracy evaluation, fourteen (14) samples were used including contrived controls (4), cell lines (2) and presumed normal CSF (8). CSF cancer specimens and FFPE samples were not included in this evaluation due to the limited availability of DNA. All samples (Fig. 1E and F) demonstrated concordant results establishing the sensitivity of Vantage^™^ directly from sheared DNA to be 100 % with a specificity of 100 %. Overall, the combined analytical sensitivity of Vantage^™^ across both methods of evaluation was demonstrated to be 95.5 % with a specificity of 100 %.

### Analytical precision – repeatability and reproducibility

Every sample processed for the Vantage^™^ assay directly or via the Summit^™^ workflow during validation was run in duplicate with different operators processing different runs, demonstrating 100 % repeatability for intra-run concordance/precision. Additionally, thirty unique specimens processed through Vantage^™^ both independently and via the Summit^™^ workflow were run by two independent operators on different days demonstrating 100 % reproducibility (inter-run precision).

## Discussion

While recent studies have proposed biomarkers such as *CDK2* [23] and *SCN3B* [24] as potential diagnostic and prognostic biomarkers for glioma patients, *MGMT* promoter hypermethylation has been shown to be a strong, independent prognostic biomarker in patients with GBM, particularly in elderly patients [19,25]. More importantly, *MGMT* methylation status is a predictive marker for response to temozolomide, making it an important biomarker to be evaluated [9,10]. In this study we present the analytical validation of Vantage^™^, the first commercially available CSF based liquid biopsy (LB) test to evaluate *MGMT* promoter methylation using enzymatic conversion of DNA instead of bisulfite conversion, minimizing DNA damage.

Belay’s Vantage^™^ assay evaluates 12 of the 98 CpG islands within the *MGMT* promoter region (Fig. 1A), one of three regions in the MGMT promoter known to be highly methylated and one of the two regions known to repress transcription significantly [26]. The assay was validated to be performed either directly from CSF derived tDNA enabling the rapid determination of sensitivity to TMZ [27] (3 day TAT) or via Summit^™^, a multi-analyte genomic profiling LB assay which also informs detection of additional gene variants, chromosome arm level alterations [20] in addition to *MGMT* promoter methylation status [27]. The combined analytical sensitivity and specificity of Vantage^™^ across both workflows was demonstrated to be 95.5 % and 100 %, respectively. Analytical sensitivity of an *MGMT* test is dependent upon a number of factors, including but not limited to specimen type, CpG sites and methodology used for evaluation with varied sensitivity [19] with the highest concordance for methylation (78 %) being shown in frozen tissues using pyrosequencing [28]. The lowest input for the assay from either workflow was demonstrated to be 5 ng, a significant improvement on current tissue requirements of 40–250 ng. Importantly, the experiments described in the current study utilized 2–3 ml of CSF, far less than that used by standard of care cytology and flow cytometry, allowing incorporation into existing clinical workflows.

Being able to perform Vantage^™^ as part of the Summit^™^ workflow from a single low input DNA CSF based assay offers tremendous advantages particularly in posttreatment GBM, wherein accurate differentiation of pseudo progression from true tumor progression (TP) represents a significant unmet clinical need. Moreso, as erroneous interpretation can lead to premature discontinuation of an effective treatment or overestimation of the efficacy of subsequent salvage therapies [29]. The capability of being able to evaluate genomic and epigenetic markers in a single assay for early recognition of TP offers the possibility for earlier therapeutic interventions, such as re-resection or recruitment to experimental clinical trials, at a time in the disease course when patients are healthier overall and with relatively preserved performance status.

## Conclusion

In this study we present the analytical validation of Vantage^™^, a novel CSF based LB assay for the evaluation of *MGMT* promoter methylation status using low input (≥5 ng) of tumor DNA from 2 to 3 mL of CSF, enabling minimally invasive, robust detection of an important biomarker for treatment and management of GBM. The novelty of the assay includes enzymatic conversion of DNA limiting degradation and fragmentation of DNA, enhancing downstream processing and the ability to perform this assay directly from CSF derived DNA (3-day TAT) or as part of Summit^™^, a multi-analyte genomic profiling assay (7–10 business day TAT).

## Figures and Tables

**Table 1:** Clinical specimen cohort with vantage^™^ results.

Clinical Samples with no cancer or known diagnosis or suspicion of Cancer			
Validation ID	*MGMT* Promoter Methylation Status	Histology Diagnosis	Vantage^™^ Result
CSF1	Methylated	Astrocytoma WHO Grade I	Unmethylated
CSF2	Unmethylated	Hydrocephalus	Unmethylated
CSF3	Unmethylated	Chiari Malformation	Unmethylated
CSF4	Unmethylated	Metastatic Melanoma	Unmethylated
CSF5	Unmethylated	Diffuse Midline Glioma, Grade 4	Unmethylated
CSF6	Unmethylated	Glioblastoma	Unmethylated
CSF7	Unmethylated	Glioblastoma	Unmethylated
CSF8	Unmethylated	Trigeminal Neuralgia	Unmethylated
CSF9	Unmethylated	Glioblastoma	Unmethylated
CSF10	Unmethylated	Glioblastoma	Unmethylated
FFPE1	Unmethylated	Choroid plexus papilloma	Unmethylated
FFPE2	Methylated	Glioblastoma	Methylated
FFPE3	Methylated	Glioblastoma	Methylated
FFPE4	Methylated	Astrocytoma	Methylated
FFPE5	Methylated	Oligodendroglioma	Methylated
FFPE6	Methylated	Pilocytic astrocytoma	Methylated
**Presumed Normal Samples**			
LC 1–38	Unmethylated	Presumed Normal	All 38 - Unmethylated

## Data Availability

Data will be made available on request.
